# Enhancing the productivity of ryegrass at elevated CO_2_ is dependent on tillering and leaf area development rather than leaf-level photosynthesis

**DOI:** 10.1093/jxb/eraa584

**Published:** 2020-12-14

**Authors:** Charilaos Yiotis, Jennifer C McElwain, Bruce A Osborne

**Affiliations:** 1 School of Biology and Environmental Science, University College Dublin, Belfield, Dublin, Ireland; 2 UCD Earth Institute, University College Dublin, Belfield, Dublin, Ireland; 3 Department of Botany, School of Natural Sciences, Trinity College Dublin, College Green, Dublin, Ireland; 4 Brookhaven National Laboratory, USA

**Keywords:** Gas exchange, growth chambers, high CO_2_, intraspecific variation, *Lolium hybridum*, *Lolium multiflorum*, *Lolium perenne*

## Abstract

Whilst a range of strategies have been proposed for enhancing crop productivity, many recent studies have focused primarily on enhancing leaf photosynthesis under current atmospheric CO_2_ concentrations. Given that the atmospheric CO_2_ concentration is likely to increase significantly in the foreseeable future, an alternative/complementary strategy might be to exploit any variability in the enhancement of growth/yield and photosynthesis at higher CO_2_ concentrations. To explore this, we investigated the responses of a diverse range of wild and cultivated ryegrass genotypes, with contrasting geographical origins, to ambient and elevated CO_2_ concentrations and examined what genetically tractable plant trait(s) might be targeted by plant breeders for future yield enhancements. We found substantial ~7-fold intraspecific variations in biomass productivity among the different genotypes at both CO_2_ levels, which were related primarily to differences in tillering/leaf area, with only small differences due to leaf photosynthesis. Interestingly, the ranking of genotypes in terms of their response to both CO_2_ concentrations was similar. However, as expected, estimates of whole-plant photosynthesis were strongly correlated with plant productivity. Our results suggest that greater yield gains under elevated CO_2_ are likely through the exploitation of genetic differences in tillering and leaf area rather than focusing solely on improving leaf photosynthesis.

## Teaser

Variability in ryegrass responses to elevated CO_2_ shows that biomass stimulation strongly associates with enhancements in leaf area/tillering capacity and is largely unrelated to leaf-level photosynthetic rate.

## Introduction

Identifying ways to enhance crop productivity remains a major goal and there are several global as well as national initiatives focused on this challenge (Bailey-Serres *et al.*, 2019). In many cases, a major target is the enhancement of leaf photosynthesis under current atmospheric CO_2_ concentrations (Long *et al.*, 2006; Evans, 2013; Foyer *et al.*, 2017; Simkin *et al.*, 2019; Wu *et al.*, 2019). The rationale for this approach is based largely on three factors: (i) modelling studies indicate that the Calvin cycle in leaves may operate at less than its optimal potential (Zhu *et al.*, 2007); (ii) both leaf photosynthesis and yield respond positively to an increase in CO_2_ concentration (Long *et al.*, 2006; Kirschbaum, 2011); and (iii) for crops where the harvest index is approaching a ceiling, the only way to further enhance yield is through an increase in leaf photosynthesis (Long *et al.*, 2006). For (i), it is unclear to what extent the results of the modelling studies can be fully exploited for yield enhancements. As far as (ii) is concerned, it is also unclear whether any inter- or intraspecific variations in the response of leaf photosynthesis to elevated CO_2_ scale directly/quantitatively with yield. Whilst recent studies have found significant intraspecific variability in leaf photosynthesis under ambient atmospheric conditions, these were not correlated with yield differences (Driever *et al.*, 2014; Faralli and Lawson, 2020; Silva-Pérez *et al.*, 2020). For (iii), this should also strictly be an increase in whole-plant photosynthesis, not leaf photosynthesis, which to a first approximation is the product of total plant leaf area and the photosynthesis of individual leaves.

A focus on leaf photosynthesis for enhancing yield has, however, met with some significant success (Simkin *et al.*, 2015; Bailey-Serres *et al.*, 2019). However, a causal relationship between leaf photosynthesis and yield may be confounded by differences in the response of individual leaves within the same canopy, not all of which show the same responses, and by parallel changes in total leaf area (Simkin *et al.*, 2015). It is important to realize that there is no *a priori* reason why an increase in leaf photosynthesis *per se* should be directly correlated with crop yield or biomass production as this will depend *inter alia* on both total plant leaf area and leaf photosynthesis (Körner, 1991). There is good evidence, for instance, that crop growth in the field is largely determined by the light-intercepting leaf area since the early studies of Monteith (Monteith and Moss, 1977). In a recent comparative study on the performance of maize and *Miscanthus*, it was shown that *Miscanthus* outperformed maize despite having a number of leaf photosynthesis attributes that were lower than those in maize because of a higher and longer duration of light-intercepting leaf area (Dohleman and Long, 2009). It has also been argued that plants may regulate photosynthesis in concert with their growth requirements (Körner, 2013; Fatichi *et al.*, 2014). This would place a greater emphasis on downstream processes, which control sink capacity and the extent to which assimilates can be converted into new biomass, as the major drivers (White *et al.*, 2016). This may be particularly important at elevated CO_2_ as a sink limitation has often been reported as a factor constraining yield increases under these conditions (Reekie *et al.*, 1998; Uddling *et al.*, 2008; Manderscheid *et al.*, 2010; Aranjuelo *et al.*, 2013; Burnett *et al.*, 2016).

Whether there are significant intraspecific variations in the response of crops to elevated CO_2_ that could be exploited is unclear. Based on a limited number of observations on ryegrass collated for the review by Poorter (1993), there might be a 50% intraspecific variation, but this is confounded by cultivar differences, and variable growth conditions and exposure times in the compiled data. Other reports also indicate contrasting responses of ryegrass to elevated CO_2_ (Ryle *et al.*, 1992; Clark *et al.*, 1995; Schenk *et al.*, 1995; Daepp *et al.*, 2001; Hager *et al.*, 2016). This argues for a better assessment of intraspecific differences in the response of crops, such as ryegrass, to elevated CO_2_ and how any variation might be exploited to enhance yields.

**Fig. 1.**
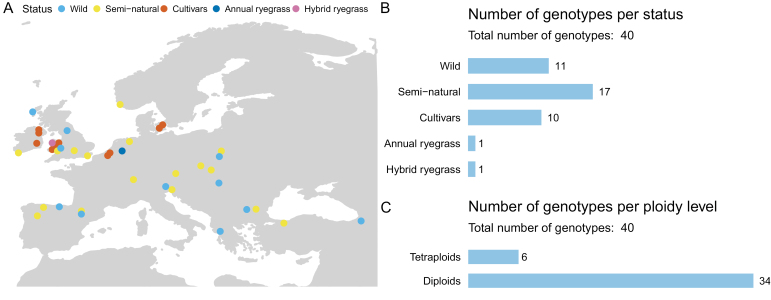
Identity of sampled genotypes. (A) Location of breeder or collection area of the cultivars and wild/semi-natural genotypes, respectively. (B) Barplot showing the numbers of cultivars, semi-natural, and wild accession of perennial ryegrass (*Lolium perenne*) used in the study. One cultivar of annual ryegrass (*Lolium multiflorum*) and a cultivar of hybrid ryegrass (*Lolium hybridum*) were also used. (C) Barplot showing the number of diploid and tetraploid genotypes used in the study.

To examine intraspecific variation in aboveground dry biomass productivity (DW) in response to elevated CO_2_ concentrations, we grew 40 genotypes of ryegrass (mostly perennial ryegrass: *Lolium perenne*) at 400 µmol mol^−1^ and 800 µmol mol^−1^ of CO_2_ in walk-in plant growth chambers (see the Materials and methods). Perennial ryegrass is the most important forage grass species in temperate agricultural grasslands, which account for 70–80% of the world’s cow’s milk, beef, and veal production (Wilkins and Humphreys, 2003). The selected genotypes comprised diploids and tetraploids, including cultivars varying in their year of introduction and wild/semi-natural plant material, collected from a wide geographic range across Europe and the Middle East (Fig. 1A–C; Supplementary Table S1). Our hypotheses were (i) that there would be significant intraspecific variations in the growth response of different ryegrass genotypes to elevated CO_2_ and (ii) based on recent evidence that these differences would be explained largely by differences in leaf photosynthesis and/or by the capacity to convert assimilates into growth.

## Materials and methods

### Plant material

Seeds from 38 perennial ryegrass, one annual ryegrass, and one hybrid ryegrass genotypes were obtained from the Institute of Biological, Environmental & Rural Sciences (IBERS) of Aberystwyth University, the Irish Department of Agriculture Food and the Marine (DAFM), and the seed company Germinal. The selection of the genotypes was made on the basis of (i) maximization of the spatial diversity of the collection areas of the semi-natural and wild accessions; (ii) inclusion of significant numbers of cultivars, semi-natural, and wild genotypes in the sample; and (iii) incorporation of diploid and tetraploid cultivars originating from a diverse range of grass breeding institutes/companies (Fig. 1A–C; Supplementary Table S1). Five out of 12 cultivars used in the study are included in the 2020 list of varieties recommended by the Irish Agriculture and Food Development Authority (Teagasc). Supplementary Table S1 provides a full list of the study varieties and details about their area of collection/breeder, ploidy, status, and provider.

### Controlled-environment experiments

Seeds from the 40 genotypes were sown, germinated, and grown concurrently for 12 weeks in four Conviron BDW-40 walk-in plant growth chambers (Conviron, Winnipeg, MB, Canada) at the Programme for Experimental Atmospheres and Climate (PÉAC) facility of University College Dublin. For each genotype, 30 seeds were sown in 10 square 4 litre pots (i.e. three seeds per pot, pot dimensions: 16 cm×16 cm×23 cm) containing John Innes No.2 Potting-on Compost. Five of the pots were then split between two chambers running a current ambient CO_2_ (ambient) atmospheric treatment, while another five were split between two chambers running a ‘2100 CO_2_’ (high CO_2_) treatment based on IPCC RCP6.0 (Pachauri *et al.*, 2014). Overall, 100 pots representing all 40 genotypes were placed and randomly mixed in each of the four growth chambers used in the study. Thus, our design consisted of (i) two treatments; (ii) four growth chambers nested in the treatments; and (iii) 100 pots per chamber representing 40 genotypes (i.e. genotypes are crossed with the chambers and treatments) for a total of 400 pots used in the study. Seed germination was marked on the 10th, 15th, and 20th day after sowing. Upon completion of germination, only the best-established seed was maintained in each pot for the remainder of the duration of the experiment. The spacing between plants in adjacent pots in the chambers was 16 cm, which is within the range of row distances used for ryegrass cultivation in the field (Koeritz *et al.*, 2015). The atmospheric compositions of the ambient and high CO_2_ treatments were 400 ppm CO_2_/21% O_2_ and 800 ppm CO_2_/21% O_2_, respectively. CO_2_ in each chamber was monitored by a WMA-4 infrared analyser (PP-Systems, Amesbury, MA, USA), and injection of compressed CO_2_ (BOC Gases Ireland Ltd, Bluebell, County Dublin, Ireland) enabled stable within-chamber CO_2_ concentrations well above ambient levels. The O_2_ concentration in each chamber was monitored by an OP-1 oxygen sensor (PP-Systems). Apart from the atmospheric CO_2_, growth conditions were the same for both treatments. Plants were grown under a 16 h/8 h simulated day/night program: 05.00–06.00 h, dawn; 06.00–09.00 h, light intensity progressively rises from 300 µmol m^−2^ s^−1^ to 600 µmol m^−2^ s^−1^; 09.00–17.00 h, mid-day light intensity of 600 µmol m^−2^ s^−1^; 17.00–20.00 h, light intensity decreases from 600 µmol m^−2^ s^−1^ to 300 µmol m^−2^ s^−1^; 20.00–21.00 h, dusk. Chamber time was staggered appropriately between the four chambers for the facilitation of time-sensitive measurements in all chambers within the same day. Treatment temperature ranged from a night-time low of 15 °C to a mid-day high of 20 °C, and relative humidity was maintained at 65% throughout the day. Chamber conditions were recorded at 5 min intervals. All plants were well watered throughout the experiment, receiving progressively increasing amounts of water, which corresponded to their growth stage. To avoid within-chamber effects, plants were rotated in the chambers every 2 weeks (i.e. five times in total). The John Innes No.2 Potting-on Compost contains enough nutrients to support plant growth for 5 weeks, at which point the plants were fed with 3 g l^–1^ Osmocote Topdress FT 4-5M (Scotts Miracle-Gro Company, Marysville, OH, USA).

### Gas exchange measurements

Upon reaching the third leaf growth stage, measurements of the operational photosynthetic rate (*A*_op_) and stomatal conductance (*g*_sop_) at the average incident irradiance in the chambers (i.e. 540 µmol m^−2^ s^−1^) were taken on the fully expanded third leaf from the top of one tiller from each plant. *A*_op_ values were also used for subsequent estimates of water use efficiency (WUE_op_). The measurements were taken between 09:00 h and 12:00 h in each chamber with a Li-Cor 6400 gas analyser (Li-Cor, Lincoln, NE, USA) equipped with a standard clear-top Li-6400 leaf cuvette and a 6400-01 CO_2_ Injector System. An MQ-200 quantum sensor (Apogee Instruments, Inc., Logan, UT, USA) was used to measure the average incident irradiance on individual leaves. Although the maximum irradiance at the canopy level was controlled at 600 µmol m^−2^ s^−1^ using the chambers’ built-in sensors, which are positioned perpendicularly relative to the direction of the main light source, individual leaves grow at different angles, thus the actual irradiance reaching the leaf surface is slightly lower. We found little variation in the irradiance reaching the leaves within and between the cabinets. The average *in situ* incident irradiance at the leaf level was 540±5 µmol m^−2^ s^−1^ (*n*=100 measurements, 25 per chamber). The Li-Cor 6400 gas analyser was set up in each growth chamber at a height where the irradiance reaching the cuvette was identical to the independently measured average *in situ* incident irradiance and then the plants were moved and positioned appropriately so that their leaves could be clamped. During the measurements, block temperature, humidity, and reference CO_2_ were controlled at values identical or very close to those of either the ambient or high CO_2_ treatments and the flow rate was set at 300 µmol s^−1^. Under these conditions, the average vapour pressure deficit with the leaves clamped in the cuvette was 1.04±0.12 kPa. Taking the measurements in the chambers using cuvette CO_2_ and H_2_O concentrations, which mimicked those of the chambers, minimized the CO_2_ and H_2_O concentration differences between the cuvette and the surrounding atmosphere, thus no post-measurement corrections were needed. Nevertheless, before each measurement, we made sure that the difference between the reference CO_2_ concentration of the analyser and the CO_2_ concentration of the empty and closed cuvette was ~0. Furthermore, the cuvette was regularly tested for leaks after clamping a leaf by exhaling in its vicinity and checking for fluctuations of the gas analyser readings. Since leaves only covered part of the 3 cm×2 cm measurement window, photographs of the clamped leaves were taken and were subsequently used to calculate the leaf areas using ImageJ software. Subsequently, all data were recalculated for the actual leaf areas by implementing the manufacturer’s equations. Positioning each plant, clamping a leaf, and taking a photograph of the clamped leaf typically took 3–5 min, after which three measurements were taken at 5–10 s intervals. To avoid build-up of exhaled CO_2_ in the chambers, measurements were taken from a laptop connected to the gas analyser and placed outside the chambers. Furthermore, the person taking the measurements always exhaled through the small openings on the chamber walls used for the cable connection of the gas analyser to the laptop. Upon clamping each leaf, stabilization of *A*_op_ and *g*_sop_ values only took ~1 min with no signs of *A*_op_ and *g*_sop_ adjustments to cuvette conditions after that point.

Responses of photosynthesis (*A*) to intercellular CO_2_ (*C*_i_) (*A*–*C*_i_ curves) were recorded for a subsample of 161 plants and were carried out on intact, fully expanded third leaves from 2–3 plants per genotype and treatment. Measurements were not performed on two genotypes whose seeds failed to germinate or develop normally thereafter (i.e. C2 cultivar and S16, Supplementary Tables S1, S2) and on the high CO_2_ treatment plants of the W10 genotype due to their very small size. For similar reasons, only one measurement was performed on the ambient-treated plants of genotypes C1, W5, W10, and HR and the high CO_2_-treated plants of the S3 genotype. The *A*–*C*_i_ curves were performed in a well-ventilated room using a Li-Cor 6400 gas analyser fitted with a 6400-40 Leaf Chamber Fluorometer. For each measurement, three leaves from three neighbouring tillers were carefully arranged to fully cover the measurement window with no overlapping. Measurements were taken between 09.00 h and 12.00 h to avoid potential mid-day stomatal closure. Airflow, leaf temperature, and vapour pressure deficit during the measurements were maintained at 500 cm^3^ min^−1^, 25.1±0.4 °C, and 1.1±0.1 kPa, respectively. Before each measurement, leaves were allowed to equilibrate at 400 µmol mol^−1^ CO_2_ and a saturating light intensity of 1500 µmol m^−2^ s^−1^, which was previously determined from eight preliminary light curves of randomly selected individuals. Full photosynthetic induction, as judged from three consecutive stable readings of light-saturated photosynthetic rate (*A*), stomatal conductance (*g*_s_), and photosynthetic electron transport rate (ETR), typically took ~30 min. CO_2_ concentration in the cuvette (*C*_a_) was then decreased stepwise from 400 µmol mol^−1^ to 50 µmol mol^−1^ (400, 300, 200, 100, and 50) and then increased from 50 µmol mol^−1^ to 1500 µmol mol^−1^ (50, 400, 600, 700, 800, 1000, 1200, and 1500) in 3 min steps. Close agreement between the two measurements taken at 400 µmol mol^−1^ in preliminary *A*–*C*_i_ curves indicated that exposure to low *C*_a_ did not affect the activation state of Rubisco. As a result, the second measurement at 400 µmol mol^−1^ was omitted in subsequent measurements to shorten the duration of the *A*–*C*_i_ curves. The short duration of the measurement precluded significant responses of stomatal conductance to superambient CO_2_.

All measurements were corrected for leaks following a standard protocol (Rodeghiero *et al.*, 2007). After initial inspection of each *A*–*C*_i_ curve, data points were assigned to either the Rubisco-limited or ribulose bisphosphate (RuBP) regeneration-limited phase of the *A*–*C*_i_ response. In cases where triose phosphate use limitation was evident at very high *C*_i_ and *A* (i.e. much higher than the operational *C*_i_ and *A* of the plants in the chambers), the corresponding data points were excluded from further analysis. Rubisco-limited (*A*_C_) and light-saturated RuBP regeneration-limited (*A*_J_) photosynthesis are described by the following equations (Long and Bernacchi, 2003; Sharkey *et al.*, 2007):

$$\begin{array}{l} A_{C}=V_{Cmax}\left[\frac{C_{i}-\Gamma^{*}}{C_{i}+K_{C}\left(1+O/K_{O}\;\right)}\right]-R_{d} \end{array}$$ (1)

$$\begin{array}{l} A_{J}=J_{max}\frac{C_{i}-\Gamma^{*}}{4C_{i}+8\Gamma^{*}}-R_{d} \end{array}$$ (2)

where *V*_Cmax_ is the maximum carboxylation rate of Rubisco, *J*_max_ is the maximum rate of RuBP regeneration at saturating light intensity, Г* is the photorespiratory CO_2_ compensation point, *K*_C_ and *K*_O_ are the Rubisco Michaelis constants for CO_2_ and O_2_, respectively, *O* is the partial pressure of O_2_ in the chloroplast (assumed equal to its atmospheric partial pressure), and *R*_d_ is the rate of respiration in the light. Following an approach similar to that of Sharkey *et al.* (2007), we used the solver utility of Microsoft Excel to minimize the sum of squares of the deviations between measured and predicted values of *A* by allowing the values of *V*_Cmax_, *J*_max_, and *R*_d_ to vary. The fitting procedure typically resulted in very good fits and sums of squares <1. Temperature-corrected *K*_C_, *K*_O_, and Г* values used for the fittings were taken from Bernacchi *et al.* (2002), and minor final temperature adjustments of the fitted values of *V*_Cmax_, *J*_max_, and *R*_d_ for the estimation of their values at 25 °C were performed using the equations of Bernacchi *et al*. (2001, 2003). All *in situ* gas exchange data are given in Supplementary Dataset S2.

### Leaf mass per area

Twelve weeks after sowing the seeds, one fully expanded blade (third leaf from the top) was sampled from each individual plant, giving a total of 369 sampled leaves. Leaf area of the samples was measured with an AM300 Leaf Area Meter (ADC BioScientific Ltd, Hertfordshire, UK). The leaves were subsequently put in paper sampling bags and oven-dried at 70 °C for 72 h. The weight of the dried samples was then measured with a microbalance, and the leaf mass per area (LMA) for each sample was calculated as the ratio of dry weight over leaf area.

### Aboveground dry biomass and tiller counts

Upon the completion of the 12 week growth period, all the aboveground fresh biomass of the study plants was harvested and put in large separate paper sampling bags. Due to the very large amount of harvested material, the samples were dried in a BDW-40 growth chamber running at 40 °C for 7 d. Humidity control was disabled and humidity levels in the chamber were <10% for the duration of the drying period. After the samples were dried, their aboveground dry biomass (DW) was measured with a PG5002-S balance (Mettler Toledo, Columbus, OH, USA). The number of tillers of each plant was subsequently counted using the dried material. In total, 50 212 tillers were counted for the 369 plants used in the study.

### Leaf area per tiller and whole-plant photosynthesis

Using DW, the tiller count, and LMA of each plant together with the leaf dry mass/DW ratio (i.e. aboveground leaf dry mass fraction, LMF_ab_) for perennial, annual, and hybrid ryegrass under both ambient and elevated CO_2_ conditions estimated in a separate experiment, we calculated the mean leaf area per tiller (LA_tiller_) for each of the study plants. In general, LMF_ab_ was very conserved among ryegrass genotypes (ambient mean value, 0.66; elevated CO_2_ mean value, 0.59) and only the annual ryegrass genotype displayed significantly different values (ambient mean value, 0.57; elevated CO_2_ mean value, 0.53). Furthermore, we used the measured values of *A*_op_ together with values of DW and LMA for each plant and LMF_ab_ for each species to approximate the *in situ* whole-plant rate of net carbon gain (*A*_plant_) of each study plant as:

$$\begin{array}{l} A_{plant}=A_{op}\times \frac{DW\times LMF_{ab}}{LMA} \end{array}$$ (3)

where *A*_op_ is in µmol m^−2^ s^−1^, DW in g per plant, LMF_ab_ in g g^−1^, LMA in g m^−2^, and *A*_plant_ in µmol s^−1^ per plant. It needs to be noted again that *A*_plant_ is an approximation of the true *in situ* rate of gross C uptake through photosynthesis minus losses associated with respiration, as its calculation assumes that all leaves of each plant receive an irradiance equal to the previously measured average irradiance at the surface of grass leaves (i.e. 540 µmol m^−2^ s^−1^) and photosynthesize at a rate equal to that measured on one mature leaf of each plant (i.e. *A*_op_).

### C:N analysis

Part of the harvested material was used for C:N analysis. Between five and 10 fully extended blades per plant were sampled and ground to fine powder using an MM200 mixer mill (Retsch, Haan, Germany). The C (C_mass_) and N (N_mass_) content per unit mass of the leaf samples were measured with a CE 440 elemental analyser (Exeter Analytical, Coventry, UK) at the microanalytical laboratory of the School of Chemistry of University College Dublin.

### Data analysis

**Fig. 2.**
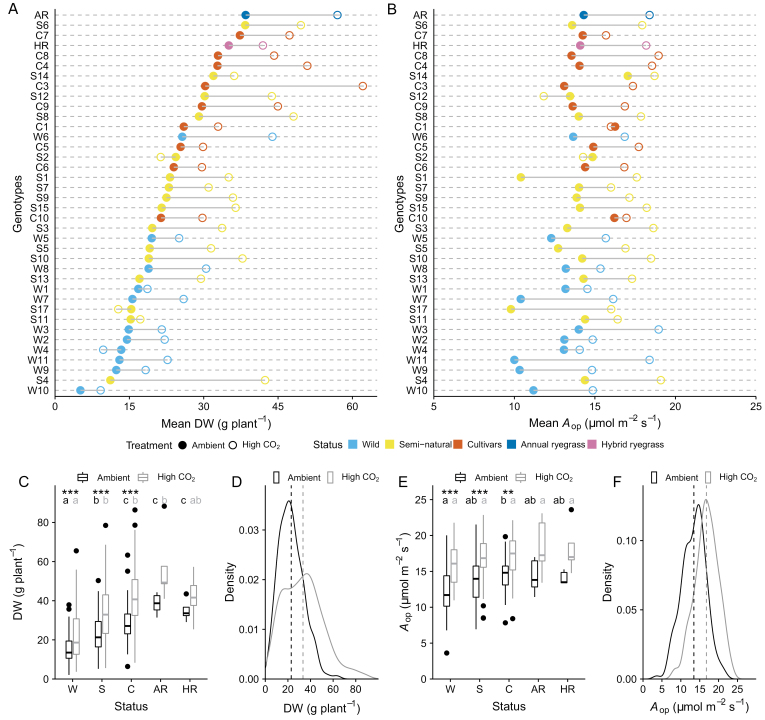
*A*_op_ and DW data comparisons. (A) Aboveground dry biomass productivity (DW) and (B) operational photosynthetic rate at average incident light intensity values under current ambient (filled circles) and high CO_2_ (open circles) conditions for 38 ryegrass genotypes. Grey lines indicate the difference in the values between the two treatments. Different colours represent different accession status or species. Boxplots of pooled DW (C) and *A*_op_ (E) data under current ambient (black boxes) and high CO_2_ (grey boxes) conditions for wild (W), semi-natural (S), and cultivated (C) genotypes of perennial ryegrass, annual ryegrass (AR), and hybrid ryegrass (HR). Boxplots illustrate the 25% and 75% quartiles (top and bottom of box) and median values (horizontal bar). Whiskers extend to the lowest and highest values that are within 1.5× interquartile range between the 25% and 75% quartiles. Black dots are statistical outliers. Asterisks denote statistically significant differences between current ambient and high CO_2_ values within each group (*P≤0.05, **P≤0.01, ***P≤0.001). Different black and grey letters signify between-group statistically significant differences (*P*≤0.05) under current ambient and high-CO_2_ conditions, respectively. Kernel density plots of measured DW (D) and *A*_op_ (F) data under current ambient (black lines) and high CO_2_ (grey lines) conditions. Dashed lines indicate the mean values for each treatment.

Data analysis was performed in R version 3.6.3 (R Core Team, 2020). Linear mixed-effects models (package ‘lme4’, Bates *et al.*, 2015) and robust linear mixed-effects models (package ‘robustlmm’, Koller, 2016) were used for trait comparisons between treatments. In the models, ‘treatment’ and ‘chamber’ were set as fixed factors (i.e. ambient and high CO_2_) and ‘genotype’ was set as a random effect factor. Although ‘chamber’ is conceptually a random effect, we opted to fit it as a fixed effect, as nesting effects with a low number of groups makes the estimation of group-level variance difficult (Schielzeth and Nakagawa, 2013). Since likelihood ratio tests (e.g. ANOVA) are not available for robust linear mixed models, *F*-tests were avoided for consistency. Instead, the estimated *t*-values and Satterthwaite approximation for degrees of freedom (Luke, 2017) were used to evaluate the significance of the marginal effect of ‘treatment’. When the residuals of a mixed-effects model violated the normality and/or homoscedasticity assumption, a robust linear mixed-effects model was fit. The *t*-value of the robust mixed-effects model output and the Satterthwaite-approximated degrees of freedom from the equivalent regular mixed-effects model were used to assess the statistical significance of the least-squares means differences (Geniole *et al.*, 2019). When the variance component of ‘genotype’ was ~0, a linear regression was fit with ‘treatment’ and ‘chamber’ as the predictor variables. In cases where the residuals of a linear regression were heteroscedastic, a heteroscedasticity-consistent variance–covariance matrix was used (MacKinnon and White, 1985) and robust *t*-values and *P*-values were calculated using the ‘lmtest’ package (Zeileis and Hothorn, 2002). For the comparisons between statuses in Fig. 2C–F, we used mixed-effects models with ‘status’ and ‘chamber’ as fixed effects and random effects from ‘genotype’. Post-hoc multiple pairwise comparisons of least squares means were performed with the ‘multcomp’ package (Hothorn *et al.*, 2008), and estimated *P*-values were adjusted for multiple comparisons with the Benjamini and Hochberg method, which controls the false discovery rate (Benjamini and Hochberg, 1995). Statistical differences in the germination rates between treatments and chambers were assessed with Fisher’s exact test.

To investigate the relationships between traits under either current ambient or high CO_2_, we used mixed-effects analysis of covariance (ANCOVA) with the ‘lme4’ and ‘robustlmm’ packages. Apart from the dependent variable and the predictor covariate, the models included ‘chamber’ as a fixed factor and random effects from ‘genotypes’. The fitted relationships and confidence limits for each treatment were extracted with the ‘effects’ package (Fox and Weisberg, 2018, 2019) and were used for plotting. *P*-values for the robust mixed-models’ predictors were calculated from the robust models’ *t*-values and Satterthwaite-approximated degrees of freedom of the equivalent regular mixed-effects models. Variance components for the fixed factors, random factors, and residuals were also extracted and used to calculate marginal *R*^2^ values of the fitted relationships (Nakagawa and Schielzeth, 2013). Potential treatment-related differences in the relationships between different traits were assessed with mixed-effects models, which additionally included ‘treatment’ as a fixed factor and its interaction with the covariate. A similar approach, using ‘status’ instead of ‘treatment’ as a fixed factor interacting with either tiller count or LA_tiller_, was used to compare status-related differences in the relationships between DW and tiller count and DW and mean LA_tiller_.

The connectivity, silhouette width, and Dunn index metrics were applied on the standardized genotype×trait matrices of means to identify the appropriate clustering method and the optimal number of clusters under ambient and high CO_2_ conditions (ciValid package, Brock *et al.*, 2008). Since trait means per genotype were used, *A*_sat_, *V*_Cmax_, *J*_max_, and *R*_d_ were excluded from the analysis as they were only measured on a subsample of the study plants. *g*_sop_ and N_mass_ were also excluded as they displayed collinearity with WUE_op_ and the C:N ratio, respectively. Hierarchical clusterings were based on Euclidean distances and the application of Ward’s minimum variance criterion. For the trait comparisons between clusters, cluster data were tested for normality and homoscedasticity using Shapiro–Wilk and Levene’s test, respectively. Normally distributed data with equal variances were analysed using Student’s *t*-test, while the Welch’s *t*-test was used for normal data with unequal variances. Non-parametric data were analysed using Mann–Whitney U-test.

Only data from plants with complete datasets were used for the Spearman’s rank correlation coefficient-based correlograms and the analysis of the different measured traits’ predictive power for DW in Fig. 5. The first step of the predictive power analysis was to run a multiple linear regression with DW as the dependent variable and the rest of the traits as predictor variables to check if the data meet the multiple linear regression assumptions. Variance inflation factor and tolerance statistics indicated the existence of collinearity between (i) C:N ratio and N_mass_; (ii) *A*_sat_, *V*_Cmax_, and *J*_max_; and (iii) *g*_sop_ and WUE_op_. Since this violates one of the assumptions of multiple regressions, N_mass_, *A*_sat_, *J*_max_, and *g*_sop_ were excluded from subsequent analysis of the traits’ predictive power. LA_tiller_ was also excluded from the analysis as it was calculated indirectly from DW, LMA, and LMF_ab_. Subsequently, we ran a multiple linear regression with the remaining predictor variables to estimate their standardized coefficients. Durbin–Watson (D–W) tests (‘car’ package, Fox and Weisberg, 2019) indicated that there is little correlation among residuals (ambient, D–W statistic=1.58; high CO_2_, D–W statistic=1.68). Studentized Breusch–Pagan tests (‘lmtest’ package) showed that the variance of the residuals is constant (ambient, *P*=0.351; high CO_2_, *P*=0.953). Inspection of the qqplots and Lilliefors tests (‘nortest’ package, Gross and Ligges, 2015) also showed that the residuals were normally distributed (ambient, *P*=0.245; high CO_2_, *P*=0.084). All Cook’s distance values were well under 1, suggesting that individual cases were not influencing the model excessively. For the neat analysis, we ran the multiple linear regression multiple times, each time removing one of the predictor variables. The *R*^2^ values of the full regression and the partial regressions were then used to estimate the increase in *R*^2^ that each trait produces when it is added to a model that already contains all the other traits.

## Results

Germination data are summarized in Supplementary Table S2. A germination rate of 75% was found for seeds sown in the chambers running the current ambient CO_2_ treatment, whilst it was 73% in the high CO_2_ treatment chambers within 20 d after sowing, resulting in a relatively high overall germination rate of 74%. Germination rates were also consistent between chambers, all of which showed rates between 71% and 75%. Observed differences in the germination rates between treatments (χ ^2^=0.844, *P*=0.358) and between chambers (χ _3_^2^=2.045, *P*=0.563) were not statistically significant. Nevertheless, germination rates 20 d after sowing varied substantially between genotypes, ranging between 3% and 100% (Supplementary Table S2).

Growth under elevated CO_2_ resulted in significant increases in DW, tiller count, and LA_tiller_ across genotypes (Table 1). Measured values of *A*_op_ and *A*_sat_ also displayed significant increases despite the high CO_2_-induced photosynthetic down-regulation evident in the significant decreases in *V*_Cmax_ and *J*_max_. An ~20% decrease in *g*_sop_ was also statistically significant and, combined with the increase in *A*_op_, resulted in a substantial 58% increase in WUE_op_ (Table 1). Plants grown at high CO_2_ also displayed significantly lower leaf N_mass_ and higher C_mass_ and C:N ratios relative to plants grown at current ambient CO_2_, while observed changes in *R*_d_ and LMA were not statistically significant (Table 1). Although the direction of the response of the different traits to high CO_2_ was not uniform among genotypes (e.g. Fig. 2A, B), the overall direction of the biomass, and photosynthetic and biochemical responses across genotypes, was in agreement with previously reported changes across species in meta-analyses of free-air CO_2_ enrichment (FACE) and controlled-environment elevated CO_2_ experiments (Ainsworth and Long, 2005; Leakey *et al.*, 2009).

**Table 1.** Summary of measured traits and differences between treatments

Parameter	Treatment	*n*	Mean	SD	CI	*t*-value	*P*	Sig.
DW (g per plant)	Ambient	185	23.0	10.8	21.4–24.5	6.7	<0.001	***
	High CO_2_	184	33.4	17.5	30.8–35.9			
*A*_op_ (μmol m^−2^ s^−1^)	Ambient	185	13.5	3.2	13.0–13.9	6.5	<0.001	***
	High CO_2_	184	16.8	3.1	16.3–17.2			
*A*_sat_ (μmol m^−2^ s^−1^)	Ambient	80	20.3	3.4	19.6–21.1	3.3	0.001	**
	High CO_2_	81	23.3	5.6	22.1–24.6			
*V*_Cmax_ (μmol m^−2^ s^−1^)	Ambient	80	71.1	12.5	68.4–73.9	–4.1	<0.001	***
	High CO_2_	81	59.4	13.5	56.4–62.4			
*J*_max_ (μmol m^−2^ s^−1^)	Ambient	80	134.3	24.4	128.8–140.0	–4.0	<0.001	***
	High CO_2_	81	112.8	26.8	106.8–118.7			
*R*_d_ (μmol m^−2^ s^−1^)	Ambient	80	0.89	0.25	0.83–0.94	–1.7	0.085	NS
	High CO_2_	81	0.84	0.28	0.78–0.90			
*g*_sop_ (mol m^−2^ s^−1^)	Ambient	185	0.32	0.14	0.30–0.34	–3.9	<0.001	***
	High CO_2_	184	0.26	0.11	0.24–0.27			
WUE_op_ (μmol mmol^−1^)	Ambient	185	5.0	1.4	4.8–5.2	12.3	<0.001	***
	High CO_2_	184	7.9	2.7	7.5–8.3			
N_mass_ (%)	Ambient	183	4.9	0.6	4.8–5.0	–5.2	<0.001	***
	High CO_2_	181	4.7	0.6	4.6–4.8			
C_mass_ (%)	Ambient	183	38.7	1.5	38.5–38.9	2.3	0.020	*
	High CO_2_	181	39.1	1.6	38.8–39.3			
C:N ratio	Ambient	183	8.0	1.2	7.8–8.2	5.1	<0.001	***
	High CO_2_	181	8.6	1.4	8.4–8.8			
LMA (g m^−2^)	Ambient	185	30.9	7.5	29.8–32.0	0.2	0.836	NS
	High CO_2_	184	31.8	9.9	30.4–33.3			
Tiller count	Ambient	183	131	51	123–138	2.3	0.025	*
	High CO_2_	180	146	65	137–156			
LA_tiller_ (m^2^ per tiller)	Ambient	183	0.0040	0.0016	0.0038–0.0042	3.1	0.002	**
	High CO_2_	180	0.0045	0.0018	0.0042–0.0048			

Number of observations (*n*), mean, SD, 95% confidence intervals (CI) of measured values under ambient and high CO_2_ conditions, *t*-values, *P-*value, and significance (Sig.) of the differences between treatments for aboveground dry mass productivity (DW), operational photosynthetic rate at growth CO_2_, and mean incident light intensity (*A*_op_), light-saturated photosynthetic rate at growth CO_2_ (*A*_sat_), maximum rate of Rubisco carboxylation (*V*_Cmax_), maximum rate of RuBP regeneration (*J*_max_), respiration in the light (*R*_d_), operational stomatal conductance (*g*_sop_), and water use efficiency (WUE_op_) at growth CO_2_ and mean incident light intensity, leaf nitrogen (N_mass_) and carbon (C_mass_) content per unit leaf dry mass, C:N ratio, leaf mass per area (LMA), number of tillers (Tiller count), and mean leaf area per tiller (LA_tiller_). Asterisks signify statistical significance at the *0.05, **0.01, and ***0.001 levels. NS, not significant.

**Fig. 3.**
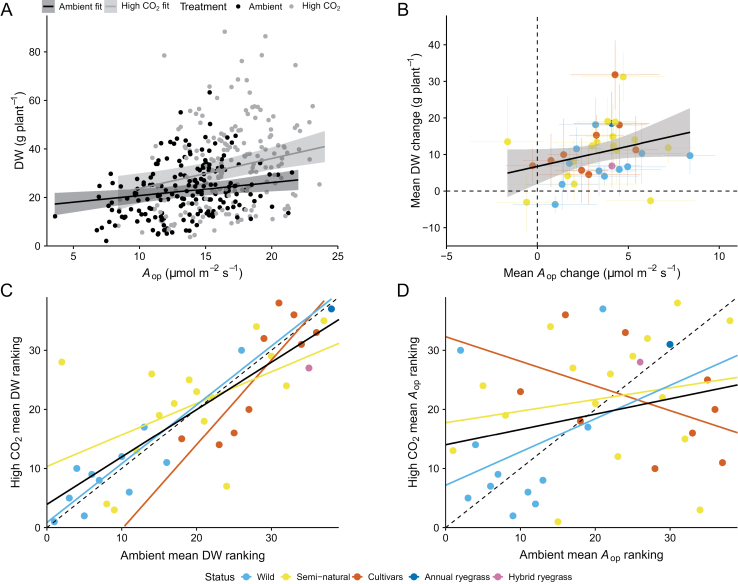
Relationships between *A*_op_ and DW values. (A) Mixed model-derived relationships (lines) and 95% confidence levels (shaded bands) between aboveground dry biomass productivity (DW) and operational photosynthetic rate at average incident light intensity (*A*_op_) under current ambient (black line, *y*=15.26 + 0.54*x*, *P*=0.006, marginal *R*^2^=0.04) and high CO_2_ (grey line, y=11.57 + 1.22x, *P*<0.001, marginal *R*^2^=0.05) conditions. Black and grey filled circles represent measured values from individual plants grown under current ambient and high CO_2_, respectively. (B) Relationship (solid line) and 95% confidence levels (shaded band) between mean changes in DW and *A*_op_ across genotypes under high CO_2_ relative to current ambient CO_2_ values (*y*=6.73 + 1.11*x*, *P*=0.070, *R*^2^=0.09). Dashed reference lines denote zero change in either *A*_op_ or DW. Data points are means ±SE per genotype, and different colours represent different accession status or species. Correlations between ranked mean DW (C) and *A*_op_ (D) data under current ambient and high CO_2_ conditions (pooled DW data, black solid line, *R*^2^=0.63, Spearman’s rank correlation coefficient=0.80; pooled *A*_op_ data, black solid line, *R*^2^=0.09, Spearman’s rank correlation coefficient=0.29). Data points in (C) and (D) represent current ambient and high CO_2_ rankings based on mean values per genotype, and different colours denote different accession status or species. Relationships for each group of genotypes are also shown. All relationships in (C) are significant (cultivars, *P*=0.004; wild, *P*<0.001; semi-natural, *P*=0.04; pooled data, *P*<0.001), while all relationships in (D) are non-significant (cultivars, *P*=0.22; wild, *P*=0.35; semi-natural, *P*=0.48; pooled data, *P*=0.12). The dashed 1:1 line is added for reference.

Significant ~7-fold variations in mean DW were found at elevated CO_2_ (Fig. 2A), which largely matched the variation found under ambient CO_2_, with a similar ranking for the genotypes under both conditions (Fig. 3C). There were, however, significant differences in the trajectory of the response of individual genotypes to CO_2_, with three genotypes showing a decrease in DW (Fig. 2A; Supplementary Fig. S1A, C). Mean DW responses ranged from –27% to +280% depending on genotype (Supplementary Fig. S1C). Changes in *A*_op_ in response to elevated CO_2_ (Fig. 1B; Supplementary Fig. S1B) varied from –12% to +84%, with a mean response of +26.5% relative to ambient values (Supplementary Fig. S1D). Of the three genotypes that displayed small decreases in *A*_op_, only one showed a decrease in DW (Fig. 2A, B). When the genotypes are grouped according to their category status (Supplementary Table S1), the significant differences observed in terms of DW are all, apart from one case (i.e. cultivars versus wild genotypes under current ambient CO_2_), not accompanied by significant differences in *A*_op_ (Fig. 2C, E). Whilst the perennial ryegrass, annual ryegrass, and hybrid ryegrass cultivars generally showed a higher DW (Fig. 2C), some semi-natural genotypes had comparable mean DW under both current ambient and elevated CO_2_ (Fig. 2A). Notably, the S6 semi-natural genotype showed the highest DW among all perennial ryegrass genotypes under ambient conditions, although its *A*_op_ fell in the mid-range of observed values (Fig. 2A, B). In general, variations in DW, particularly at high CO_2_, were greater than the corresponding variations in *A*_op_ (Fig. 2A–C, E), with evidence of a more heterogeneous response of DW in the elevated CO_2_ treatment (Fig. 2D, F).

Assessment of the traits underpinning these variations indicated that *A*_op_ was a poor predictor of DW under both ambient and high CO_2_ conditions (Fig. 3A). Whilst the relationship between *A*_op_ and DW was statistically significant under both ambient (*P*=0.006) and high CO_2_ (*P*<0.001), *A*_op_ could only resolve 4% and 5% of the variation in DW values, respectively (current ambient CO_2_, marginal *R*^2^=0.04; high CO_2_, marginal *R*^2^=0.05). Mean DW responses to elevated CO_2_ were also poorly correlated with corresponding responses in *A*_op_ (Fig. 3B, *P*=0.07, *R*^2^=0.09). Examination of the ranking of genotypes in terms of their DW at ambient CO_2_ with their ranking at elevated CO_2_ showed a highly positive relationship, whilst there was little evidence that *A*_op_ under ambient conditions was related to those under elevated CO_2_ conditions (Fig. 3C, D).

**Fig. 4.**
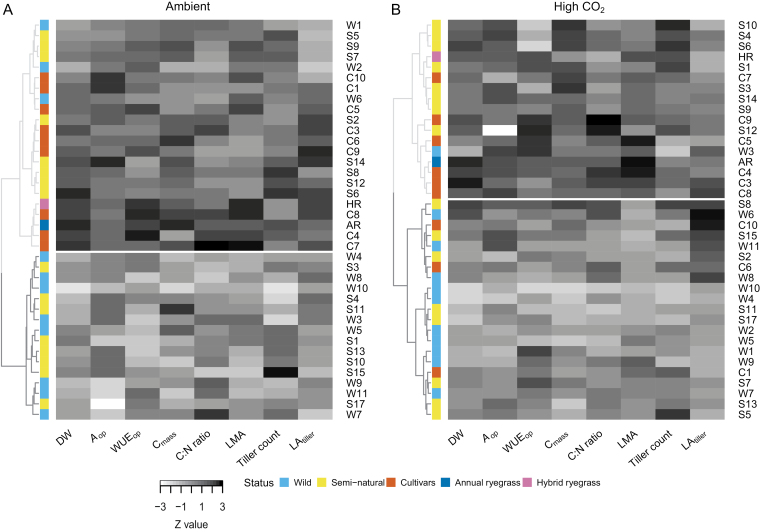
Hierarchical clusterings of genotypes under current ambient and high CO_2_. Heatmaps and *Z*-score hierarchical clusterings of genotypes based on Euclidean distances and the application of Ward’s minimum variance criterion for a range of morphological and physiological traits under current ambient (A) and high CO_2_ (B). Rows represent the 38 ryegrass genotypes in the study, and columns represent the traits used for the clustering. The different colours on the vertical colour bar designate the accession status or species of the corresponding genotype. Horizontal white lines separate the two identified clusters in both (A) and (B).

Two clusters of genotypes were identified under both ambient and high CO_2_ conditions, based on a range of morphological and physiological traits associated with photosynthesis and growth (Fig. 4A, B). However, the genotypic composition of the ambient and high CO_2_ clusters differed. Under ambient conditions, all cultivars, eight semi-natural genotypes, and three wild genotypes were grouped (Fig. 4A), forming a cluster, which, with the exception of the C:N ratio, had significantly higher trait values compared with the second cluster comprising the remaining semi-natural and wild genotypes (Table 2). Under elevated CO_2_ conditions, cultivars, semi-natural, and wild genotypes were grouped in both identified clusters (Fig. 4B). Trait comparisons showed that apart from LA_tiller_ and WUE_op_, cluster 1, which included most of the cultivars, half of the semi-natural genotypes, and one wild genotype, had significantly higher trait values compared with cluster 2 (Table 2). Since most intercluster differences were significant, the analysis did not result in a clear picture regarding the parameters most closely associated with the differences in DW under ambient and high CO_2_ conditions. Nevertheless, it showed that the wild and semi-natural genotypes can be a valuable source of variability that could potentially be exploited to enhance DW under high CO_2_.

**Table 2.** Trait comparisons between clusters

	Ambient			High CO_2_		
	Cluster 1 (*n*=22)	Cluster 2 (*n*=16)		Cluster 1 (*n*=17)	Cluster 2 (*n*=21)	
Parameters	Mean±SD	Mean±SD	Sig.	Mean±SD	Mean±SD	Sig.
DW (g plant^−1^)	27.67±6.74	16.16±4.6	***	41.69±9.83	25.96±10	***
*A*_op_ (μmol m^−2^ s^−1^)	14.21±1.08	12.39±1.93	*	17.62±1.79	16.1±1.32	**
WUE_op_ (μmol mmol^−1^)	5.35±0.59	4.36±0.58	***	8.13±1.4	7.46±0.95	NS
C_mass_ (%)	39.06±0.66	38.13±0.74	***	40.03±0.69	38.42±0.97	***
C:N ratio	8.13±0.72	7.72±0.68	ns	8.94±0.9	8.19±0.65	**
LMA (g m^−2^)	32.94±3.96	27.94±2.43	***	37.21±6.47	27.37±4.04	***
Tiller count	140±21.18	115.78±41.39	*	162.57±42.8	131.73±40.39	*
LA_tiller_ (m^2^ per tiller)	0.0043±0.001	0.0036±0.0005	*	0.0043±0.0008	0.0045±0.0014	NS

Mean ±SD values of the parameters used in the hierarchical clustering for each of the clusters identified under current ambient and high CO_2_. *P-*values and significance for the observed intercluster differences are also included (*P≤0.05, **P≤0.01, ***P≤0.001. NS, not significant).

**Fig. 5.**
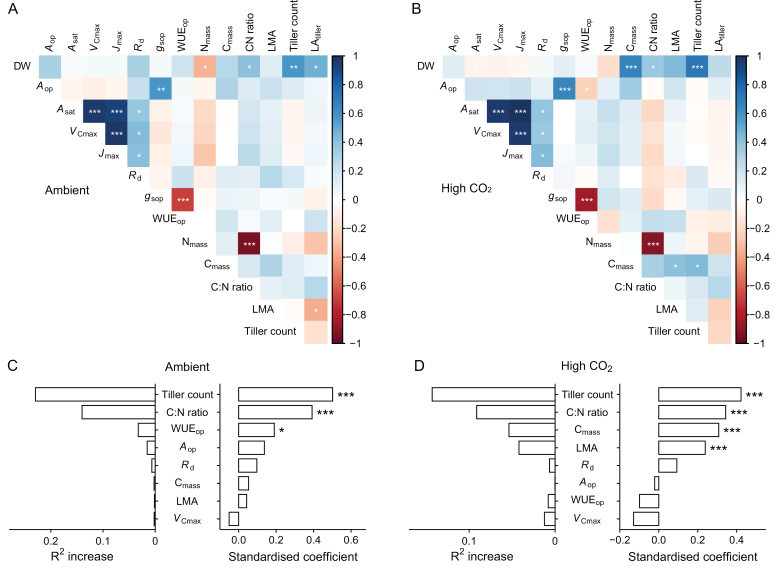
Trait correlations and predictive power. Spearman’s rank correlation coefficient-based correlograms of traits measured on plants grown under current ambient (A) and high CO_2_ (B). The colour of each square indicates the value of the correlation coefficient for each pair of traits following the colour scale of the vertical colour bar. Asterisks are plotted for the correlations whose *P*-value is *≤0.05, **≤0.01, or ***≤0.001. Standardized coefficients of the parameters used in the multiple linear regressions with DW as the dependent variable and neat analysis results (i.e. increase in *R*^2^ that each trait produces when it is added to a model that already contains all of the other traits) for the current ambient (C) and high CO_2_ (D) datasets. Asterisks denote statistically significant relationships between the corresponding independent variables and DW at the *0.05, **0.01, or ***0.001 levels. Only data from plants with complete datasets were used for the analysis.

Spearman’s rank correlation coefficient-based correlograms of the measured parameters emphasized the strong correlation between tiller count (current ambient and elevated CO_2_) and LA_tiller_ (current ambient CO_2_) and DW (Fig. 5A, B). For N_mass_ under current ambient conditions, C_mass_ under high CO_2_, and the C:N ratio under both conditions, there was also a strong correlation with DW. Importantly, there is little evidence that *A*_op_ or *A*_sat_ correlated well with DW. The same is true for a series of physiological parameters, including *V*_Cmax_, *J*_max_, *R*_d_, *g*_sop_, WUE_op_, and LMA. To assess the most important predictive variable for DW, we ran a multiple linear regression and carried out a neat analysis (see Materials and methods) using DW as the dependent variable and the remaining variables as independent variables, after removing those indirectly calculated from DW or showing collinearity. Calculation of the standardized beta coefficients and the neat analysis (i.e. the increase in *R*^2^ that each trait produces when it is added to a model that already contains all of the other traits) showed that the tiller count is the strongest predictor for DW under both ambient and elevated CO_2_ conditions, followed by the C:N ratio. In comparison, *A*_op_ appears to have negligible predictive power (Fig. 5C, D).

**Fig. 6.**
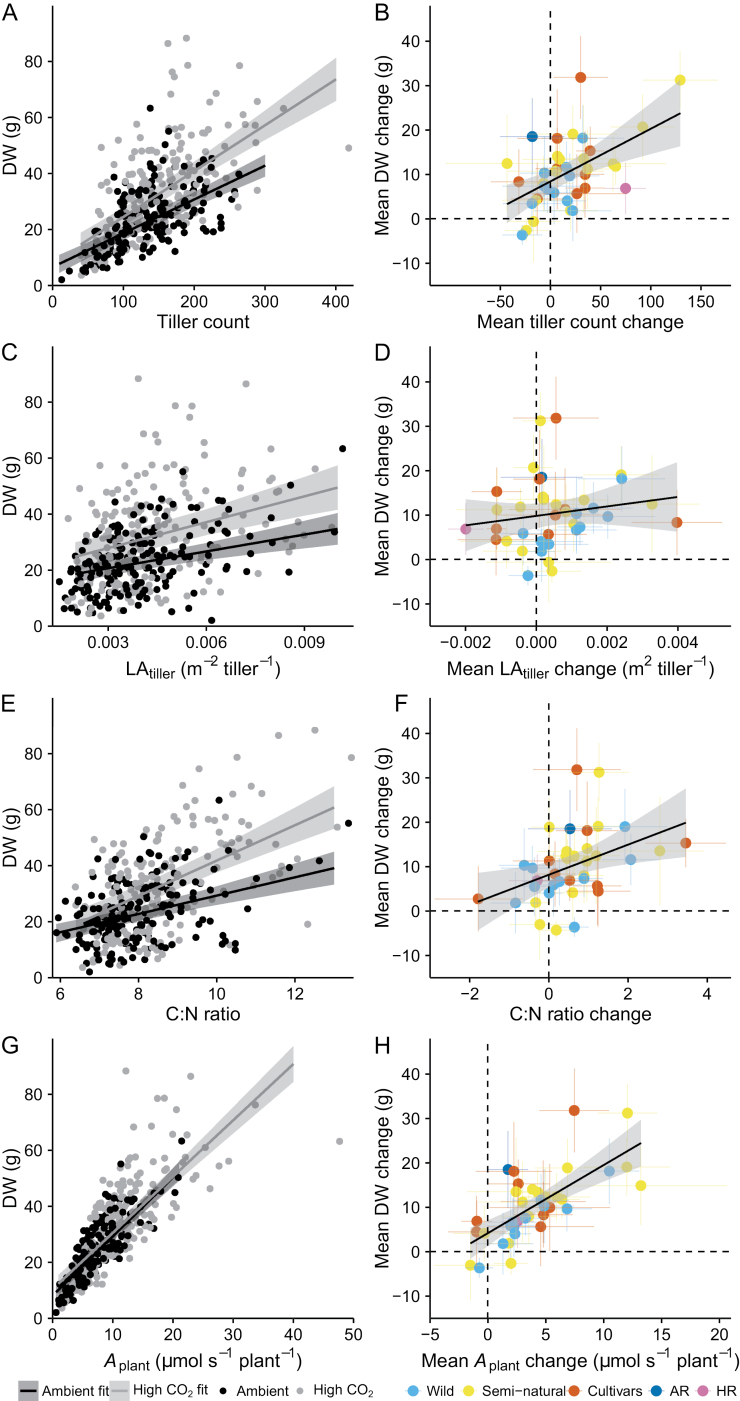
Relationships between DW and tiller count, LA_tiller_, C:N ratio, and *A*_plant_. (A) Mixed model-derived relationships (lines) and 95% confidence levels (shaded bands) between aboveground dry biomass productivity (DW) and tiller count under current ambient (black line, *y*=0.12*x*+6.58, *P*<0.001, marginal *R*^2^=0.37) and high CO_2_ (grey line, *y*=0.16*x*+8.11, *P*<0.001, marginal *R*^2^=0.42) conditions. (B) Relationship (solid line) and 95% confidence levels (shaded band) between mean changes in DW and tiller count across genotypes under high CO_2_ relative to current ambient CO_2_ values (*y*=0.12*x*+8.46, *P*<0.001, *R*^2^=0.29). (C) Mixed model-derived relationships (lines) and 95% confidence levels (shaded bands) between DW and mean leaf area per tiller (LA_tiller_) under current ambient (black line, *y*=2006*x*+14.66, *P*<0.001, marginal *R*^2^=0.10) and high CO_2_ (grey line, *y*=3079*x*+18.63, *P*<0.001, marginal *R*^2^=0.10) conditions. (D) Relationship (solid line) and 95% confidence levels (shaded band) between mean changes in DW and LA_tiller_ across genotypes under high CO_2_ relative to current ambient CO_2_ values (*y*=1066*x*+9.85, *P*=0.311, *R*^2^=0.03). (E) Mixed model-derived relationships (lines) and 95% confidence levels (shaded bands) between DW and leaf C:N ratio under current ambient (black line, *y*=3.26*x*–3.33, *P*<0.001, marginal *R*^2^=0.14) and high CO_2_ (grey line, *y*=6.31*x*–21.30, *P*<0.001, marginal *R*^2^=0.26) conditions. (F) Relationship (solid line) and 95% confidence levels (shaded band) between mean changes in DW and leaf C:N ratio across genotypes under high CO_2_ relative to current ambient CO_2_ values (*y*=3.39*x*+8.21, *P*=0.010, *R*^2^=0.17). (G) Mixed model-derived relationships (lines) and 95% confidence levels (shaded bands) between DW and estimated *in situ* whole-plant rate of carbon gain (*A*_plant_) under current ambient (black line, *y*=2.11*x*+8.10, *P*<0.001, marginal *R*^2^=0.67) and high CO_2_ (grey line, *y*=2.01*x*+10.41, *P*<0.001, marginal *R*^2^=0.65) conditions. (H) Relationship (solid line) and 95% confidence levels (shaded band) between mean changes in DW and *A*_plant_ across genotypes under high CO_2_ relative to current ambient CO_2_ values (*y*=1.53*x*+4.22, *P*<0.001, *R*^2^=0.49). Black and grey filled circles in (A), (C), (E), and (G) represent measured values from individual plants grown under current ambient and high CO_2,_ respectively. Data points in (B), (D), (F), and (H) are means ±SE per genotype, and different colours represent different accession status or species. Dashed reference lines denote zero change.

The relationships between tiller count, LA_tiller_, C:N ratio, and *A*_plant_ with DW are further explored in Fig. 6. We found a strong positive relationship between tiller count and DW (Fig. 6A, current ambient CO_2_, *P*<0.001, marginal *R*^2^=0.37, conditional *R*^2^=0.75; high CO_2_, *P*<0.001, marginal *R*^2^=0.42, conditional *R*^2^=0.65) and poorer relationships with LA_tiller_ (Fig. 6C, current ambient CO_2_, *P*<0.001, marginal *R*^2^=0.10, conditional *R*^2^=0.51; high CO_2_, *P*<0.001, marginal *R*^2^=0.10, conditional *R*^2^=0.51) and C:N ratio (Fig. 6E, current ambient CO_2_, *P*<0.001, marginal *R*^2^=0.14, conditional *R*^2^=0.57; high CO_2_, *P*<0.001, marginal *R*^2^=0.26, conditional *R*^2^=0.55). Combining the total plant leaf area with *A*_op_ to provide an estimate of *A*_plant_ (i.e. whole-plant rate of net carbon gain) resulted in a strong positive correlation with DW (Fig. 4G, current ambient CO_2_, *P*<0.001, marginal *R*^2^=0.67, conditional *R*^2^=0.78; high CO_2_, *P*<0.001, marginal *R*^2^=0.65, conditional *R*^2^=0.80). Significant positive correlations were also obtained between the mean changes in DW from ambient to elevated CO_2_ and corresponding changes in the tiller count (Fig. 6B, *P*<0.001, *R*^2^=0.29), C:N ratio (Fig. 6F, *P*=0.01, *R*^2^=0.17), and *A*_plant_ (Fig. 6H, *P<*0.001, *R*^2^=0.49), while no significant relationship was found between mean DW and LA_tiller_ changes (Fig. 6D, *P*=0.31, *R*^2^=0.03). When only perennial ryegrass genotypes are considered, the *R*^2^ of the relationship between the mean changes in DW and tiller count increases to 0.40. In all other cases, the *R*^2^ shows marginal changes or remains unchanged.

## Discussion

In terms of our first hypothesis, we demonstrate significant variation in the DW of ryegrass genotypes at ambient and elevated CO_2_ (Fig. 2A, C) and in their DW response to elevated CO_2_, relative to ambient values (Supplementary Fig. S1C). Whilst increased leaf photosynthesis is the major focus of many current studies aimed at enhancing crop productivity, the results presented here indicate that differences in tillering (Figs 5A–D, 6A, B) and whole-plant leaf area are the major factors contributing to intraspecific differences in ryegrass aboveground productivity. In terms of our second hypothesis, DW is shown to correlate well with *A*_plant_ (Fig. 6G, H), which is expected since all the available C for growth comes from photosynthesis. However, it is the total leaf area, rather than *A*_op_ (Fig. 3A, B) that establishes this correlation between assimilated C and DW. This means that leaf area is more important in determining *A*_plant_ under either ambient or high CO_2_, and in driving changes in DW. Although we may have underestimated the contribution of leaf photosynthesis to DW in our estimates of whole-plant photosynthesis, as they were based on measurements made on single leaves, leaf area was a far more significant explanatory variable (compare Fig. 3A, B with Fig. 6G, H). Recent results have also shown that leaf area is largely responsible for interspecific differences in the productivity of switchgrass genotypes exposed to low temperature or salinity (Cordero and Osborne, 2017; Cordero *et al.*, 2019). However, a number of studies have attributed differences in productivity to modifications in leaf photosynthesis (Ort *et al.*, 2015; Simkin *et al.*, 2015; Driever *et al.*, 2017; Bailey-Serres *et al.*, 2019; South *et al.*, 2019). The results of these studies suggest that small and often age-related increases in leaf photosynthesis can result in significant yield or biomass differences (Simkin *et al.*, 2015; Driever *et al.*, 2017; South *et al.*, 2019) presumably due to the compounding effect of the leaf photosynthetic rate on biomass accumulation or yield. However, whether the reported differences in leaf photosynthesis can quantitatively account for the productivity/yield differences remains to be assessed, to the best of our knowledge. It is also clear that any observed variation in biomass production will be inextricably linked to differences in leaf area as plant biomass increases, which may confound the identification of the major driver(s) associated with variations in productivity. It is interesting that although the majority of individual genotypes displayed increases in both *A*_op_ and DW in response to elevated CO_2_ concentration (Fig. 2A, B), *A*_op_ explained little of the variability in DW observed in intraspecific comparisons (Fig. 3A). This indicates that a positive correlation between leaf photosynthetic rate and productivity for individual species or genotypes with increasing CO_2_ concentration may not be a good argument for focusing on leaf photosynthesis for enhancing productivity.

In terms of the major reason underlying the intraspecific differences in biomass production, this was clearly not due to differences in any leaf photosynthetic attributes (Fig. 5A–D). Whilst biomass production was positively correlated with C:N ratio, with high biomass/high tillering associated with high C:N ratios, this was not correlated with *A*_op_ or *V*_Cmax_, indicating that this was not related to variations in Rubisco amount and/or specific activity. This suggests that the higher C gain per unit of N with increased tillering is presumably a result of a reduction in any feedback effects on photosynthesis due to an increase in the available sinks for photoassimilates. Whilst this does not rule out leaf photosynthesis as a target for yield enhancement, the results presented in this work suggest that any gains may be small and place more of an emphasis on downstream controls on plant productivity and the capacity to convert photoassimilates into growth (Körner, 2013; Fatichi *et al.*, 2014) as well as how this might be manipulated to enhance yields.

Based on the similar DW-based ranking of genotypes from the ambient and elevated CO_2_ treatments (Fig. 3C), selection for increased tillering/whole-plant leaf area at ambient CO_2_ would generally result in an improved growth at elevated CO_2_, potentially making this a more practical approach that does not require selection under higher atmospheric CO_2_ concentrations. We need to note, however, that focusing on a few genotypes performing well under ambient conditions, rather than exploiting the overall variability to select for important traits, would be risky. The cluster analysis highlighted that the relative performance of a genotype can change depending on the growth CO_2_ concentration. As a result, some genotypes clustered with either the low-producing or high-producing genotypes, depending on the growth CO_2_ concentration. A notable example is the semi-natural genotype S4, which showed the second lowest DW under ambient conditions (Fig. 2A), yet under elevated CO_2_ it displayed the highest increase in tiller count and the second highest increase in DW (Fig. 6B; Supplementary Fig. S1C, D), and clusters together with the highest-producing cultivars (Fig. 4B). It could be argued that the trait estimates for S4 might have been affected by the genotype’s relatively low germination rate under current ambient CO_2_; however, other genotypes with high germination rates also displayed very large or atypical differences between CO_2_ treatments (e.g. genotypes C3, S2, S17, and W4; Fig. 2A; Supplementary Table S2).

The observed relationships between DW at ambient or high CO_2_ and tiller count/LA_tiller_ provide evidence of similar trajectories for both the cultivars and the wild or semi-natural genotypes, indicating that natural selection, as well as plant breeding, have increased DW in the same way (Supplementary Fig. S2A, B). Also, the similar DW-based ranking of the genotypes at ambient and high CO_2_ (Fig. 3C) suggests that differences in the response to high CO_2_ have been driven mainly by differences in their potential growth/tillering responses and are not a result of CO_2_ acting directly as a selection agent.

Overall, these results suggest that greater gains in grass aboveground productivity may be possible through manipulations in tillering/whole-plant leaf area rather than through manipulations in leaf photosynthesis. Increased tillering/LA_tiller_ also results in more rapid canopy closure following defoliation or during establishment, and could enhance productivity by increasing the amount of light intercepted over the growing season. Burnett *et al.* (2016) also suggested that developing new sinks, such as those provided by tillers, alleviates high CO_2_-induced sink limitations. In contrast, Tausz-Posch *et al.* (2015) have reported that a wheat genotype with high tillering capacity did not benefit more from high CO_2_ compared with a genotype with low tillering capacity. The large genetic variation in tillering-related yield differences reported in this and other studies (Mathan *et al.*, 2016; Xie *et al.*, 2016) indicates that this is an important target for selection. A consideration of previously reported biomass and tillering capacity responses of perennial ryegrass genotypes to high CO_2_ reveals a mixed picture. For example, Ryle *et al.* (1992), Clark *et al.* (1995), Schenk *et al.* (1995), and Hager *et al.* (2016) report no significant changes in ryegrass tiller numbers under high CO_2_, although Ryle *et al.* (1992) and Schenk *et al.* (1995) observed an increase in shoot biomass. In contrast, Daepp *et al.* (2001) observed increases in both tillers and productivity during vegetative growth. Increases in tiller number under high CO_2_ have also been reported for other species such as wheat (Hocking and Meyer, 1991) and rice (Kobayashi *et al.*, 2006). Our results show that the apparent discrepancies between previous reports of ryegrass DW and tiller count responses to high CO_2_ can be accounted for by high intraspecific variability. This highlights the limitations of focusing on a single or a few genotypes when testing for high CO_2_ effects on ryegrass or other crops and, due to the strong correlation between the two parameters, offers an easily tractable trait to use for breeding highly productive crops for the future.

In terms of realizing any potential differences in tillering or leaf area development, there is a surprising lack of information on the factors that regulate tillering under natural conditions (Parsons and Chapman, 2000; Laidlaw, 2004). The use of individual spaced potted plants in the growth room experiments may have facilitated tillering to a greater extent than might be seen in densely packed swards in the field. Grazing or defoliation, depending on its timing and severity, can also have significant impacts on tillering (Gastal and Lemaire, 2015; Yuan *et al.*, 2020), as can fertilizer applications (Lee *et al.*, 2017). This argues for a more judicious examination of the appropriate management practice and planting density, together with a consideration of canopy architectural differences, that may be required to optimize light capture, resource use, and biomass production under field conditions (Venuto *et al.*, 2004; Deng *et al.*, 2012; Warnasooriya and Brutnell, 2014; Xie *et al.*, 2016; Yang *et al.*, 2019). Given that competition for soil resources is the most likely explanation for biomass–density relationships in plant stands (Weiner and Freckleton, 2010), improvements in the use of water and nutrients may be particularly important for realizing any potential gains associated with an increase in tillering/leaf area.

The translation of these results to other crops such as cereals, where only a portion of the plant biomass is allocated to harvestable yield (harvest index, HI), may not be straightforward. The report by Tausz-Posch *et al.* (2015) may suggest that a high tillering capacity might not benefit the response of wheat to high CO_2_. However, given the high intraspecific variability shown in this study, this could reflect, at least in part, cultivar differences. The data in Fig. 6B demonstrate that a higher capacity for tiller formation under elevated CO_2_ does not always translate into a higher DW response. Interestingly increased tillering has been linked to yield increases in wheat and spring barley (Granger, 2016; Xie *et al.*, 2016). Based on the argument that higher yields may only be possible by increasing whole-plant productivity because any further increases in HI are limited (Long *et al.*, 2006), increases in tillering/leaf area may also be significant targets for selection and this will be aided by new insights into the factors that control branching and plant architecture (Kebrom *et al.*, 2013; Tavakol *et al.*, 2015).

### Conclusions

Considering future increases in the world’s population, the need for corresponding increases in crop productivity has become a top priority. In this work, we demonstrate significant natural and man-made genetic variability in the performance of 38 genotypes of ryegrass, the most important grass species used in temperate agriculture, to elevated CO_2_ concentrations. We further show that variations in productivity under both current and elevated CO_2_ concentrations is primarily linked to traits associated with tillering and plant leaf area rather than leaf photosynthetic traits. Not only does our study provide a genetically tractable target for breeding high CO_2_-ready perennial ryegrass cultivars, it also highlights the importance of preserving natural genetic variation for utilization in future crop breeding programmes.

## Supplementary data

The following supplementary data are available at *JXB* online.

Table S1. Details and codes of the genotypes used in the study.

Table S2. Germination data.

Fig. S1. DW and *A*_op_ data for each genotype under current ambient and high CO_2_, and relative responses of DW and *A*_op_ to high CO_2_.

Fig. S2. Slope comparisons of the relationships between DW and tiller count and between DW and LA_tiller_ for cultivars, semi-natural, and wild perennial ryegrass genotypes.

Dataset S1. Mean trait values for the genotypes used in the study.

Dataset S2. *In situ* gas exchange data for the genotypes used in the study.

eraa584_suppl_Supplementary_Materials

eraa584_suppl_Supplementary_Datasets

## Data Availability

Mean trait values for the genotypes used in the study can be found in the provided Supplementary Dataset S1. The raw data supporting the findings of this study are available from the corresponding author (Charilaos Yiotis) upon reasonable request.
