# Supplementary material for: Enhancing the productivity of ryegrass at elevated CO2 is dependent on tillering and leaf area development rather than leaf-level photosynthesis
Source: J Exp Bot. 2020 Dec 14;72(5):1962–77. doi: 10.1093/jxb/eraa584 (PMC7921301; doi:10.1093/jxb/eraa584)
Supplement: eraa584_suppl_Supplementary_Materials [file eraa584_suppl_supplementary_materials.pdf]

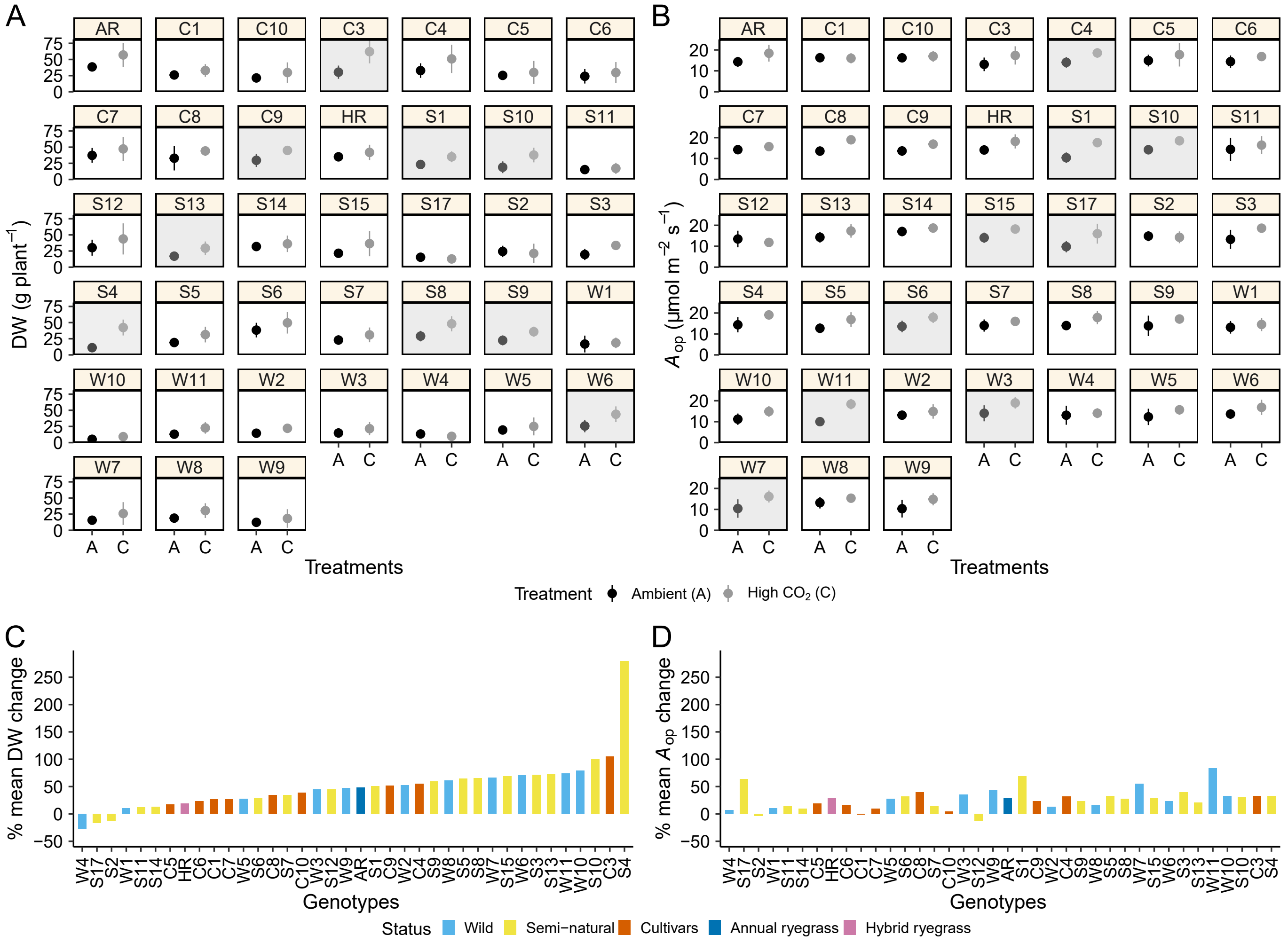

Fig. S1. DW and A_{op} data for each genotype under current ambient and high CO_2 and relative responses of DW and A_{op} to high CO_2. (A) Mean±SD values of aboveground dry biomass productivity (DW) and (B) operational photosynthetic rate at average incident light intensity (A_{op}) per ryegrass genotype under current ambient (A: black) and high CO_2 (C: grey) conditions. Grey panels indicate statistically significant differences ($P<0.05$). (C) Ascending order of % changes in DW under high CO_2 relative to current ambient conditions. Different colours represent different accession status or species. (D) % changes in A_{op} under high CO_2 relative to current ambient conditions. The sequence of genotypes follows the ascending order in (C).

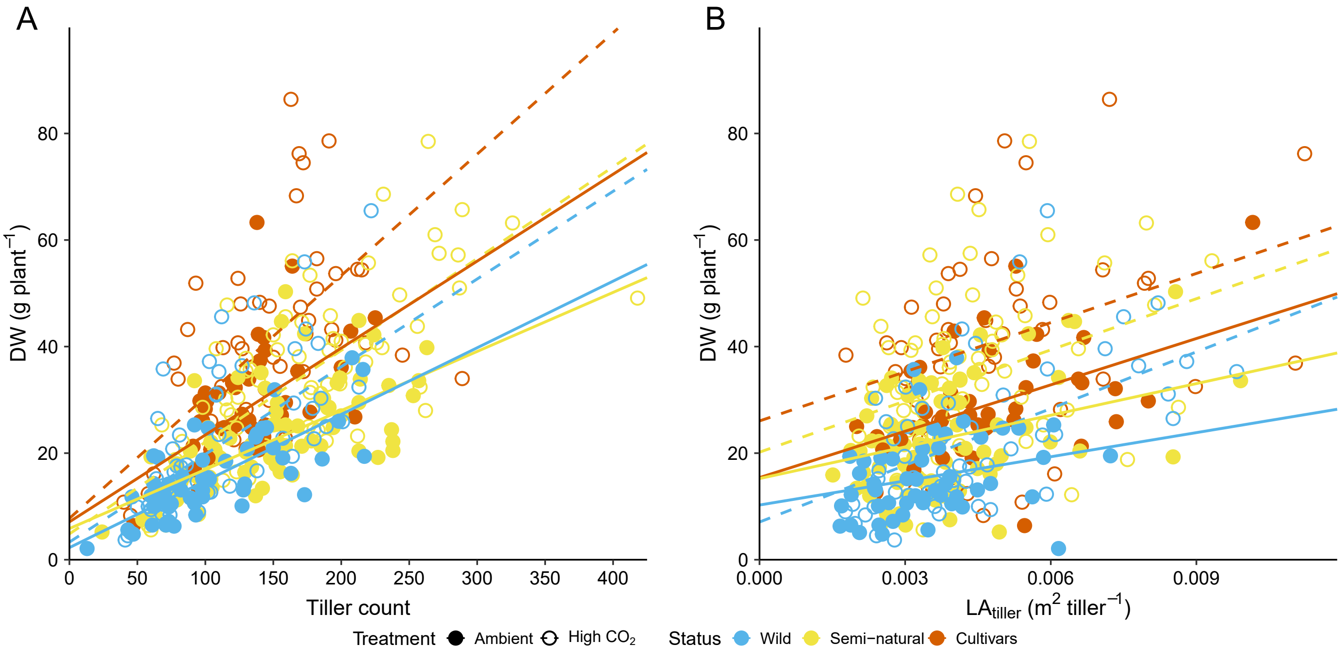

Fig. S2. Slope comparisons of the relationships between DW and tiller count and between DW and LA_{tiller} for cultivars, semi-natural and wild perennial ryegrass genotypes. (A) Mixed model-derived relationships between aboveground dry biomass productivity (DW) and tiller count under current ambient (solid lines) and high CO_2 (dashed lines) for perennial ryegrass cultivars (tawny), semi-natural (yellow) and wild (light blue) genotypes. Closed and open circles represent measured values from individual plants grown under current ambient and high CO_2, respectively. ANOVA showed no significant 'Tiller count×Status' interaction under both current ambient ($F_{(2, 146)}$=2.30, P=0.104) and high CO_2 ($F_{(2, 149)}$=1.45, P=0.238) conditions. (B) Mixed model-derived relationships between DW and mean leaf area per tiller (LA_{tiller}) under current ambient (solid lines) and high CO_2 (dashed lines) for perennial ryegrass cultivars (tawny), semi-natural (yellow) and wild (light blue) genotypes. Closed and open circles represent measured values from individual plants grown under current ambient and high CO_2, respectively. ANOVA showed no significant 'LA_{tiller}×Status' interaction under both current ambient ($F_{(2, 161)}$=0.77, P=0.467) and high CO_2 ($F_{(2, 162)}$=0.06, P=0.944) conditions.

Table S1. Details and codes of the genotypes used in the study. Names of cultivars and accession numbers of wild and semi-natural genotypes, codes used throughout the manuscript, ploidy level, breeder or collection area, years of introduction or collection and seed providers for the genotypes used in the study.

Name or accession number	Status	Code	Species	Ploidy	Breeder/Collection area	Introduction/ collection	Provider
Moy	Cultivar	C1	*Lolium perenne*	Diploid	AFBI, N. Ireland	1980's	IBERS
Talbot	Cultivar	C2	*Lolium perenne*	Diploid	VDH BV, Netherlands	1980's	IBERS
Boyne	Cultivar	C3	*Lolium perenne*	Diploid	DLF, Denmark	2013	DAFM
Carraig	Cultivar	C4	*Lolium perenne*	Tetraploid	Teagasc, Ireland	2012	DAFM
Dunluce	Cultivar	C5	*Lolium perenne*	Tetraploid	AFBI, N. Ireland	2007	DAFM
S23	Cultivar	C6	*Lolium perenne*	Diploid	IBERS, Wales	1930's	IBERS
Aberchoice	Cultivar	C7	*Lolium perenne*	Diploid	IBERS, Wales	2003	DAFM
Abergain	Cultivar	C8	*Lolium perenne*	Tetraploid	IBERS, Wales	2006	DAFM
Aspect	Cultivar	C9	*Lolium perenne*	Tetraploid	DLF, Denmark	2014	DAFM
Vigor	Cultivar	C10	*Lolium perenne*	Diploid	ILVO, Belgium	1960's	IBERS
Ba 10015	Semi-natural	S1	*Lolium perenne*	Diploid	Port Meadow, England	1977	IBERS
Ba 10103	Semi-natural	S2	*Lolium perenne*	Diploid	Bryne, Norway	1981	IBERS
Ba 10153	Semi-natural	S3	*Lolium perenne*	Diploid	Roaringwater Bay, Ireland	1981	IBERS
Ba 10282	Semi-natural	S4	*Lolium perenne*	Diploid	Mümliswil, Switzerland	1982	IBERS
Ba 10292	Semi-natural	S5	*Lolium perenne*	Diploid	Rye, England	1982	IBERS
Ba 10985	Semi-natural	S6	*Lolium perenne*	Diploid	Eferding, Austria.	1986	IBERS
Ba 11427	Semi-natural	S7	*Lolium perenne*	Diploid	Bialobrzegi, Poland.	1990	IBERS
Ba 11862	Semi-natural	S8	*Lolium perenne*	Diploid	Valasske Mezirici, Czechia	1992	IBERS
Ba 11887	Semi-natural	S9	*Lolium perenne*	Diploid	Banska Bystrica, Slovakia	1992	IBERS
Ba 12028	Semi-natural	S10	*Lolium perenne*	Diploid	Stara Reka, Bulgaria	1993	IBERS
Ba 13099	Semi-natural	S11	*Lolium perenne*	Diploid	Bragança, Portugal	1995	IBERS
Ba 13448	Semi-natural	S12	*Lolium perenne*	Diploid	Ronchi dei Legionari, Italy	1998	IBERS
Ba 13698	Semi-natural	S13	*Lolium perenne*	Diploid	Broto, Spain	2001	IBERS
Ba 13867	Semi-natural	S14	*Lolium perenne*	Diploid	Pola de Lena, Spain	2003	IBERS
Ba 9151	Semi-natural	S15	*Lolium perenne*	Diploid	Adapazari, Turkey	1971	IBERS
Ba 9246	Semi-natural	S16	*Lolium perenne*	Diploid	Winschoten, Netherlands	1972	IBERS
Ba 9799	Semi-natural	S17	*Lolium perenne*	Diploid	Llanddeusant, Wales	1979	IBERS
Ba 11311	Wild	W1	*Lolium perenne*	Diploid	Szarvas, Hungary	1988	IBERS
Ba 11429	Wild	W2	*Lolium perenne*	Diploid	Kielce, Poland	1990	IBERS
Ba 11900	Wild	W3	*Lolium perenne*	Diploid	Dodoni, Greece	1992	IBERS
Ba 12019	Wild	W4	*Lolium perenne*	Diploid	Troyan, Bulgaria	1993	IBERS
Ba 13228	Wild	W5	*Lolium perenne*	Diploid	Alston, England.	1996	IBERS
Ba 13445	Wild	W6	*Lolium perenne*	Diploid	Claut, Italy.	1998	IBERS
Ba 13697	Wild	W7	*Lolium perenne*	Diploid	Broto, Spain.	2001	IBERS
Ba 13861	Wild	W8	*Lolium perenne*	Diploid	La Vega, Spain.	2003	IBERS
Ba 14025	Wild	W9	*Lolium perenne*	Diploid	St Kilda, Scotland.	2005	IBERS
Ba 9123	Wild	W10	*Lolium perenne*	Diploid	Ardanuç, Turkey.	1971	IBERS
Ba 9791	Wild	W11	*Lolium perenne*	Diploid	Aberystwyth, Wales.	1979	IBERS
Fabio	Cultivar	AR	*Lolium multiflorum*	Tetraploid	DSV, Germany.	1998	Germinal
AberEve	Cultivar	HR	*Lolium hybridum*	Tetraploid	IBERS, Wales.	2004	Germinal

Table S2. Germination data. Number of seeds sowed and germination rates on days 10, 15 and 20 after sowing at the treatment, chamber and genotype levels. Day 20 germination rates per genotype for each of the two treatments are also shown.

			Germination rate (%)				
		Seeds sowed	Day 10	Day 15	Day 20	Day 20/Ambient	Day 20/High CO_2
– Treatments	Ambient	600	67	72	75		
	High CO_2	600	64	71	73		
– Chambers	Ambient 1	300	66	72	75		
	Ambient 2	300	65	73	74		
	High CO_2 1	300	63	69	71		
	High CO_2 2	300	68	72	75		
– Genotypes	C1	30	50	67	70	73	67
	C2	30	0	0	10	13	7
	C3	30	80	83	83	80	87
	C4	30	83	83	87	93	80
	C5	30	73	87	90	87	93
	C6	30	33	43	53	53	53
	C7	30	87	90	93	100	87
	C8	30	80	80	80	87	73
	C9	30	93	93	93	100	87
	C10	30	17	57	63	60	67
	S1	30	80	87	87	93	80
	S2	30	63	70	70	73	67
	S3	30	27	37	37	67	7
	S4	30	20	37	40	20	60
	S5	30	87	97	97	93	100
	S6	30	90	90	90	93	87
	S7	30	47	53	53	53	53
	S8	30	43	53	57	40	73
	S9	30	33	50	53	60	47
	S10	30	60	63	63	53	73
	S11	30	33	37	37	33	40
	S12	30	93	93	93	93	93
	S13	30	77	90	93	93	93
	S14	30	97	97	97	93	100
	S15	30	73	73	73	73	73
	S16	30	0	3	3	7	0
	S17	30	53	63	73	67	80
	W1	30	83	90	93	100	87
	W2	30	80	80	80	87	73
	W3	30	93	100	100	100	100
	W4	30	83	83	83	73	93
	W5	30	47	53	53	67	40
	W6	30	83	83	87	87	87
	W7	30	93	93	93	93	93
	W8	30	93	97	97	100	93
	W9	30	90	90	90	93	87
	W10	30	80	83	87	100	73
	W11	30	50	73	77	80	73
	AR	30	83	83	83	80	87
	HR	30	80	87	87	87	87
